# Novel Candidates for Vaccine Development Against *Mycoplasma Capricolum* Subspecies *Capripneumoniae* (Mccp)—Current Knowledge and Future Prospects

**DOI:** 10.3390/vaccines7030071

**Published:** 2019-07-23

**Authors:** Mohd Iqbal Yatoo, Oveas Raffiq Parray, Muheet, Riyaz Ahmed Bhat, Qurat Un Nazir, Abrar Ul Haq, Hamid Ullah Malik, Mujeeb Ur Rehman Fazili, Arumugam Gopalakrishnan, Shah Tauseef Bashir, Ruchi Tiwari, Sandip Kumar Khurana, Wanpen Chaicumpa, Kuldeep Dhama

**Affiliations:** 1 Mycoplasma Laboratory, Division of Veterinary Clinical Complex, Faculty of Veterinary Sciences and Animal Husbandry, Jammu and Kashmir, Srinagar 190006, India; 2 Department of Veterinary Clinical Medicine, Madras Veterinary College, Tamilnadu Veterinary and Animal Sciences University, Vepery 600007, India; 3 Department of Molecular and Integrative Physiology, University of Illinois, Urbana-Champaign, Urbana, IL 61801, USA; 4 Department of Veterinary Microbiology and Immunology, College of Veterinary Sciences, Deen Dayal Upadhayay Pashu Chikitsa Vigyan Vishwavidyalay Evum Go-Anusandhan Sansthan (DUVASU), Mathura 281001, India; 5 ICAR-Central Institute for Research on Buffaloes, Sirsa Road, Hisar 125001, India; 6 Center of Research Excellence on Therapeutic Proteins and Antibody Engineering, Department of Parasitology, Faculty of Medicine Siriraj Hospital, Mahidol University, Bangkok 10700, Thailand; 7 Division of Pathology, ICAR-Indian Veterinary Research Institute, Izatnagar, Bareilly 243122, India

**Keywords:** *capripneumoniae*, contagious caprine pleuropneumonia, genomics, *Mycoplasma*, proteomics, vaccines

## Abstract

Exploration of novel candidates for vaccine development against *Mycoplasma capricolum* subspecies *capripneumoniae* (Mccp), the causative agent of contagious caprine pleuropneumonia (CCPP), has recently gained immense importance due to both the increased number of outbreaks and the alarming risk of transboundary spread of disease. Treatment by antibiotics as the only therapeutic strategy is not a viable option due to pathogen persistence, economic issues, and concerns of antibiotic resistance. Therefore, prophylactics or vaccines are becoming important under the current scenario. For quite some time inactivated, killed, or attenuated vaccines proved to be beneficial and provided good immunity up to a year. However, their adverse effects and requirement for larger doses led to the need for production of large quantities of Mccp. This is challenging because the required culture medium is costly and *Mycoplasma* growth is fastidious and slow. Furthermore, quality control is always an issue with such vaccines. Currently, novel candidate antigens including capsular polysaccharides (CPS), proteins, enzymes, and genes are being evaluated for potential use as vaccines. These have shown potential immunogenicity with promising results in eliciting protective immune responses. Being easy to produce, specific, effective and free from side effects, these novel vaccine candidates can revolutionize vaccination against CCPP. Use of novel proteomic approaches, including sodium dodecyl sulfate polyacrylamide gel electrophoresis (SDS-PAGE), two-dimensional gel electrophoresis, immunoblotting, matrix-assisted laser desorption/ionization-time-of-flight (MALDI-TOF) mass spectrometry, tandem mass spectroscopy, fast protein liquid chromatography (FPLC), bioinformatics, computerized simulation and genomic approaches, including multilocus sequence analysis, next-generation sequencing, basic local alignment search tool (BLAST), gene expression, and recombinant expression, will further enable recognition of ideal antigenic proteins and virulence genes with vaccination potential.

## 1. Introduction

*Mycoplasma capricolum* subspecies *capripneumoniae* (Mccp) is the causative agent of contagious caprine pleuropneumonia (CCPP), which is a potentially devastating transboundary contagious disease endangering the goat population in more than 40 countries [1,2,3,4,5]. Conventional vaccines against this disease, such as live-attenuated, killed/inactivated whole cells [6,7,8,9], sonicated bacteria [10], or saponin-based adjuvant preparations [11,12], have several limitations, including a large dose requirement, production constraints (purity, quality, and cost), efficacy, and innocuousness that have hampered their wide distribution and practical usage [2,13]. Therefore, novel vaccine candidates are being sought to prevent and control the disease in the future [4,5,13,14]. Using new technologies, which include proteomic approaches, sodium dodecyl sulfate polyacrylamide gel electrophoresis (SDS-PAGE) [15], matrix-assisted laser desorption/ionization-time-of-flight (MALDI-TOF) mass spectrometry [16], tandem mass spectroscopy [13], fast protein liquid chromatography (FPLC) [17], immunoblotting [18], and genomic approaches, such as multilocus sequence analysis (MLSA), next-generation sequencing (NGS), basic local alignment search tool (BLAST), gene expression [19] and recombinant protein production [16], several potential vaccine antigens have been identified, such as capsular polysaccharides [15], membranous or cellular proteins (heat shock protein 70 (HSP70), variable surface proteins (Vsps), transketolase, elongation factor G, cytosol aminopeptidase family catalytic domain protein, aldehyde dehydrogenase (NAD) family protein, thioredoxin reductase (NADPH), elongation factor Tu (Ef-Tu), and the peptidase M24 family) [13,16,17,20]. Other candidates include genes involved in coding of structural units, such as membrane proteins, or antigens (the *glpF*, *glpK*, and *glpD* gene cluster and the *gtsA*, *gtsB*, and *gtsC* gene cluster) [17,21], enzymes involved in physiological or metabolic pathways, such as the pyruvate dehydrogenase complex (PDHC), L-α-glycerophosphate oxidase (GlpO), transketolase, phosphoenolpyruvate protein phosphotransferase, glutamyl-tRNA amidotransferase subunit A, L-lactate dehydrogenase [16,17,19,20], as well as other candidates known to be involved in the bacterial pathogenicity and metabolic pathways, including glycerol metabolism and hydrogen peroxide production pathways [17,19]. Some of these new candidates not only show promising roles in vaccines [13,16,19], but also facilitate rapid identification of the causative agent (Mccp) [15,22]. However, accurate identification, immunological evaluation, and effectiveness of these entities need proper investigation prior to their field application in livestock [5,14]. The present review provides details regarding advanced aspects of CCPP vaccine development, including prophylactics used in the past, current research, and future prospects, with brief insights into novel technologies that have been applied.

## 2. Need for CCPP Vaccines

CCPP is a respiratory infectious disease that causes significant economic loss to goat farmers [4,5,23]. Approximately 507 million US dollars are lost yearly in endemic areas due to morbidity, mortality, and loss of production, in addition to costs involved in the prevention, control, and treatment [4,14]. Recent emergence of CCPP in newer areas, like Afghanistan [24], Mauritius [22], Tajikistan [25], Pakistan [26], India [4,5], China [27,28], Saudi Arabia [29], and Qatar [30], as well as increased incidence of outbreaks in prevalent areas like Ethiopia [31], Kenya [32,33], Tanzania [34], Turkey [35], and the African Union [1] has also raised concerns. Outbreaks are even occurring in wild animals [36,37,38], endangering the neighboring countries as there is always a risk of spreading the disease [5,39,40]. Treatment by antibiotics as the only therapeutic strategy for CCPP is not a viable option due to the persistence of Mccp pathogens in treated animals, which act as disease carriers [5,41]. Besides, large-scale and repeated antibiotic treatments of herds cause economic constraints for farmers due to the expense and concerns for antibiotic resistance [4,5,14,27,40,42,43]. Hence, the role of prophylactics, such as vaccines, is becoming imperative under the current scenario in order to prevent CCPP outbreaks and control its spread [2,41,44]. Disease control also prevents the need for the mass culling of animals in endemic areas, problems associated with the trade of affected animals, and other non-feasible preventive measures [2,44,45].

## 3. CCPP Vaccines: Historical Perspective and Development

Initially, goats were vaccinated subcutaneously with lung extracts obtained from affected animals [46]. Attenuated high passage broth cultures of *Mycoplasma* strain F38 were initially used [47]. The practice of using pleural fluid or lung homogenates directly as crude vaccines, though proven effective, had numerous flaws [2,39]. This was changed to the utilization of the *Mycoplasma* F38 strain [6] or subcutaneous injection of live Mccp into naïve goats, a procedure which did not cause any untoward inflammatory reactions [2]. In the beginning stages of CCPP immunity studies [48,49] there was always a risk of infection associated with the use of live Mccp pathogens. Therefore, inactivated or attenuated preparations of Mccp organisms were developed [8,10,11]. Rurangirwa et al. [10] used sonicated antigens of the F38 strain of *Mycoplasma* with incomplete Freund’s adjuvant, emulsified in aluminum hydroxide and phosphate buffered saline, and found that goats immunized with this antigenic preparation developed a strong immunity. Rurangirwa et al. [11] found that 0.15 mg of F38 *Mycoplasma* in saponin was the optimal formulation for inactivated *Mycoplasma* vaccine, providing immunity for more than a year. This vaccine could be stored for 14 months at either 4 °C or 22 °C without losing its immunogenicity. King [50] used formalinized *Mycoplasma* culture as a vaccine and reported that the optimum age for vaccination of the kids should be beyond ten weeks of age since maternal antibodies are effective up to eight weeks [50]. Litamoi et al. [8] determined that 3 mg of saponin is ideal for inactivation of 1 mL of sonicated protein having a concentration of 2 mg/mL and used as an inactivated vaccine. The inactivated *Mycoplasma* strain F38-saponin vaccine showed 100% protection against natural CCPP [8]. The immune response to polysaccharide vaccines was evaluated by Rurangirwa et al. [51]. Rurangirwa et al. [52] used 0.15 mg dose of lyophilized, saponin-killed *Mycoplasma* strain F38 as a field vaccine, and noted complete protection against mortality and 95% efficacy against clinical disease. The World Organization for Animal Health (OIE) [2] has recommended this saponin-inactivated vaccine for CCPP with 0.15 mg of freeze-dried Mccp protein and 3 mg of saponin in a dose of 1 mL per goat. Such a vaccine has more than a 14-month shelf life and provides immunity for approximately 12 months. Although this vaccine is believed to be pure, safe, and effective, the larger dose of antigen, Mccp culture and production constraints, adjuvant (saponin) proinflammatory reactions, and overall cost of production have become concerns [2,5,12,40]. Moreover, the vaccine is not recommended for pregnant animals because of the possible reaction to saponin [2]. 

When considering the issues of using whole organisms, the limitations of production, along with the potential for inflammatory reactions, such as swelling at the administration site, rising body temperature after vaccination, incompatibility with other vaccines, and chances of adverse effects in pregnant animals, two perspectives have emerged. On one hand, different components or antigens of Mccp are needed for evaluation as a potential vaccine. On the other hand, various adjuvants need to be evaluated for resolving potential issues. Isolation and identification of specific antigens or components of Mccp [15,18,53,54] and evaluation of different adjuvants [12,17,19] or their concentrations [22] have begun in order to develop safe and effective vaccines. Identification of antigens by polyacrylamide gel electrophoresis (PAGE) was performed by Thiaucourt et al. [53]. Thiaucourt et al. [54] identified and isolated 112 crude or membrane proteins antigens from *Mycoplasma* strain F38 by spotting on nitrocellulose using immunobinding, standardizing protein levels to 0.5 mg/mL, and using monoclonal antibodies (mAbs) to identify four out of 60 specific antigens. This study became the basis for the development of mAb-based ELISAs for the diagnosis of Mccp [22]. However, the immunogenic potential of these antigens as vaccines is yet to be evaluated. Rurangirwa et al. [55] identified a specific integral membrane surface protein of Mccp using the mAb E8-18. The inhibitory effects of vaccines containing subunit fractions of Mccp were studied by March and Jones [56]. Capsular polysaccharides (CPS) of Mccp have been isolated and evaluated as antigens for both diagnostic [15] and prophylactic purposes [56,57]. These were utilized for the rapid detection of Mccp using the latex agglutination test [15] and their immunogenic potential has been explored for vaccine development [15,56,57]. Through immunoblotting and blocking ELISAs, March et al. [18] recognized some immunodominant core proteins (108, 70, and 62 kDa) in Mccp that can serve as recombinant proteins used as a diagnostic reagent and/or prophylactic vaccine component. Simultaneously, saponin-adjuvant vaccines were compared to MONTANIDE ISA 50 adjuvant vaccines. The latter is proven to be better in terms of safety and effectiveness and could be combined with the anthrax vaccine [12]. Aluminum hydroxide terpene vaccines have also been recommended [58]. 

In order to reduce cost, required quantities, and adverse reactions to adjuvants, studies were performed to compare live and killed Mccp vaccines. Live Mccp vaccines were found to be more effective in seroconversion (84.2%) than the killed vaccine (68.4%) [59]. Sixteen out of nineteen goats inoculated with live vaccine showed seropositivity as compared to thirteen goats out of nineteen vaccinated with killed vaccine. Lakew et al. [60] reported that 253 out of 414 (61.1%) goats were seroconverted following vaccination with inactivated vaccine and the seroconversion was higher in young adult goats compared to older ones. Tesgera et al. [61] noted 60.71% mean seropositivity out of seven and a mean percent inhibition of 61.52% out of eight in inactivated whole culture vaccinated goats when compared to positive control goats, i.e., infected non-vaccinated goats (58.86% and 51.86%, respectively). In addition, there were no other adverse clinical effects. In some countries or regions, few commercial vaccines are available. They are either live (e.g., Pulmovac and Capridoll in Turkey), killed (e.g., CCPPV in Ethiopia), or inactivated (e.g., Caprivax in Kenya) [62]. Vaccines from the Kenya Veterinary Vaccines Production Institute (KEVVAPI) are an inactivated Mccp vaccine with saponin as the adjuvant [11].

A few CCPP vaccines are being manufactured in Africa by the National Veterinary Institute (NVI) in Ethiopia and KEVVAPI in Kenya. Similarly, in the Middle East vaccines are manufactured by the Jordan Bio-Industries Center (JOVAC) in Jordan, by IBRIZE in Saudi Arabia, by VETAL in Turkey, and recently attempts are being made by the Global Alliance for Livestock Veterinary Medicines (GALVmed) in China. However, there are concerns regarding the quality of these vaccines when considering European Pharmacopoeia guidelines (European Food Safety Authority Animal Health and Welfare (EFSA AHAW) [13,40]. Protein quality and quantity, *Mycoplasma* species, relative amounts of *Mycoplasma* antigens and residual proteins from the cultural medium need proper evaluation in the vaccines, especially probable *Mycoplasma* species and peptides from medium need to be detected properly [13]. There is a need for the development of more efficient and cheaper vaccines for CCPP, along with the ability to maintain the standard of quality [40].

Details of the various Mccp vaccines are shown in Table 1.

## 4. The Current State of CCPP Vaccine Development

Under the current developmental scenario, thrust is given to genomic and proteomic approaches for deciphering the genes involved in the expression of proteins or polysaccharides that form an important part of the putative antigenic or virulence factors. Although a novel approach and still in infancy, many reports have surfaced depicting a series of genes, proteins, capsular polysaccharides, and metabolic pathways having interdependence and are vital for pathogen virulence and pathogenicity of the disease. Though conventional approaches and whole pathogen-based commercial or trial vaccines are the main entities of prophylaxis as stated earlier, however limitations of their production and utilization makes realization of the genomic and proteomic approaches a necessity. Hence, a discussion on these aspects helps in better understanding of their role in CCPP vaccination possibilities.

## 5. Proteomic Approaches

Proteins (both membranous and cellular) [13,16,17], genes [17,21], enzymes or their subunits [16,19], or metabolic pathway components [17,19] are potential candidates of interest for vaccine development. Most of these biomolecules have immunogenic properties and hence possess probable vaccination potential, while others can be evaluated for the same. With advanced technologies, novel explorations are revealing newer cellular and subcellular components having immunogenic roles. MALDI-TOF spectroscopy-based proteomics revealed that nine membranous and twenty cellular proteins are antigenic in Mccp [16]. Pyruvate dehydrogenase complex (PDHC), heat shock protein 70 (HSP70), transketolase, elongation factor G, phosphoenolpyruvate protein phosphotransferase, and glutamyl-tRNA amidotransferase subunit A have shown more than 99% homology, whereas L-lactate dehydrogenase, cytosol aminopeptidase family catalytic domain protein, aldehyde dehydrogenase (NAD) family protein, thioredoxin reductase (NADPH), elongation factor Tu (Ef-Tu), and peptidase M24 family have shown more than 98% homology to proteins from related species [16]. These proteins have potent immunogenicity and hence can be novel vaccine candidates. They have roles in pathogenic mechanisms including adhesion of organism to host surfaces through pyruvate dehydrogenase, receptor binding of host and pathogen through HSP70, eliciting immune response through elongation factor Tu (Ef-Tu), and binding to mucin through glyceraldehyde-3-phosphate dehydrogenase (GapA) [16]. Some have conserved sequence, hence can be potential vaccine candidate. Among these proteins, four members of the PDHC, Ef-Tu, HSP70 and GapA are found in both cellular and membrane fractions. This all confirms the immunogenic role of membrane proteins and their abundance in the cell. However, only nine immunogenic proteins in membrane have been identified so far attributing to various technological and inherent pathogenic factors [16]. This can be better elucidated through in vivo experimentation on these immunogenic proteins evaluating humoral immune response for better understanding role in localization, colonization and immunogenicity of Mccp. Further, three proteins have been optimized and expressed, including the pyruvate dehydrogenase E1 component alpha subunit (PDHA), pyruvate dehydrogenase complex E1 component beta subunit (PDHB), and dihydrolipoyllysine-residue acetyltransferase components of the PDHC [17]. The three optimized genes were expressed in *Escherichia coli* and the relevant proteins were analyzed further by in vivo expression and antibody detection. They showed in vivo expression, immunogenicity, and an effective immune response, and thus can serve as potential vaccine candidates [17]. Variable surface proteins, which have been identified in Mccp are essential for antigenic variation, immune evasion, and act as pathogenic factors for some *Mycoplasma* species [20]. VmcC lipoprotein (XDU01000612) and P60 surface lipoprotein (XDU01000037), which may be associated with Mccp virulence [20], have roles in antigenic variation as well as survival and virulence, respectively. Hemolysin A (XDU01000067), which acts as an erythrocyte lysing virulence factor, has been identified in Mccp [20]. More than 2000 peptides have been noted in various Mccp vaccines by tandem mass spectroscopy, out of which a set of 163 and 144 different peptide sequences were identified in different Mccp samples. Of these specific peptides, 118 were common in different samples and thus believed to be potential vaccine candidates [13]. Further, in vivo expression of L-α-glycerophosphate oxidase (GlpO) has been confirmed [19]. The role of peroxides in glycerol metabolism and pathogenesis has been elucidated. These processes infer that there is a role in host–pathogen interactions during CCPP, leading to subsequent vaccine development [19]. A brief description of these protein/enzyme-based vaccine candidates is given below:

**Pyruvate dehydrogenase complex** plays an important role in glycolysis with the TCA cycle and is composed of three enzymes, including pyruvate dehydrogenase (E1), dihydrolipoyl transacetylase (E2), and dihydrolipoyl dehydrogenase (E3). These enzymes catalyze an irreversible reaction that converts pyruvate into acetyl-CoA by a process called pyruvate decarboxylation [69,70].

**HSP70** is a molecular chaperone which is remarkably preserved in all living organisms. This protein is prompted in response to stress and is associated with cellular transformation. In the cytosol, it shows some essential functions such as targeting stress-accumulated misfolded proteins, transporting proteins, supporting antigen processing and presentation, and preventing protein aggregation. Following stress, intracellularly located HSPs play defensive functions, while extracellularly located HSPs act as danger signals and elicit immune responses [71].

**Transketolase** encoded by the *TKT* gene is a thiamine-dependent enzyme. It is a key enzyme involved in the Calvin–Benson–Bassham cycle of photosynthesis and the non-oxidative part of the pentose phosphate pathway, where it converts fructose-6 phosphate into pentose-5 phosphate [72]. 

**Elongation factor G,** known as translocase, has an important role in protein translation. It is involved in two phases of protein synthesis, in the translocation step of the elongation phase, and in ribosome disassembly following termination [73].

**Phosphoenolpyruvate protein phosphotransferase** is an enzyme consisting of two substrates, including phosphoenolpyruvate and protein histidine. This enzyme is related to the transferase family and transfers phosphorus groups (phosphotransferases) [74]. 

**Lactate dehydrogenase** is an enzyme present in all living organisms. This enzyme is responsible for the conversion of lactate to pyruvate and the reverse. It converts NAD^+^ to NADH and the reverse [75].

**Cytosol aminopeptidase family catalytic domain protein** is a leucyl aminopeptidase and is mainly involved in proteolysis and protein metabolic processes. It has been noted in *Mycoplasma leachii* PG50 and has a homology of around 98.2% to *Mycoplasma capricolum* subsp. *capripneumoniae* strain M1601 [16] and 95.8% sequence identity to Mccp strain GM12. It is expressed by gene locus MSB_A0207 in Mccp [16]. It causes inactivation of host immune peptides and induces inflammatory response and hence act as virulence factor [76].

**Thioredoxin reductase** is the only enzyme known to reduce thioredoxin. The biological functions of this enzyme are cell growth, protection against oxidative stress, and the regulation of p53, a known tumor-suppressor protein and transcription factor that has been identified in various human cancers [77].

**Elongation factor Tu** promotes the catalysis and binding of aminoacyl-tRNA (AA-tRNA) to ribosomes during the elongation phase of translation during protein biosynthesis. It is one of the most plentiful and highly preserved proteins in prokaryotes [78].

**Peptidase M24 family proteins** are Xaa-Pro dipeptidases noted in *Mycoplasma leachii* PG50 and have homology of 98.9% to *Mycoplasma capricolum* subsp. *capripneumoniae* strain M1601 [16] and 91.9% sequence identity to *Mycoplasma mycoides* subsp. *mycoides* SC type, PG1 strain. They are expressed by gene locus MSB_A0355 [16]. They help in proteolysis by hydrolysis of protein bonds.

## 6. Genomic Approaches

Genomic approaches enable the identification of genes that have a potential role in coding for proteins (membranous and cytoplasmic), structural components (capsule), enzymes, or metabolic pathways that form important components of antigens or contribute to the virulence of Mccp. Both genetic and genome sequencing approaches have been undertaken to decipher genetic mapping of Mccp. Polymorphisms in the 16S rRNA genes [65] and sequencing of the H2 locus [66] and of the arginine deiminase operon [67] were followed by whole genome sequencing of many Mccp strains, including strains M1601 [21], 9231-Abomsa [79], F38, ILRI181 [80], and M1601 [20]. Polymorphisms in the 16S rRNA genes have been noted by Pettersson et al. [65]. In the Lorenzon et al. [66] study, where they sequenced the H2 locus, a 2400-bp fragment of Mccp was amplified and found to contain two duplicated genes encoding a putative membrane protein. Woubit et al. [67] identified a DNA fragment of 7109 bp containing arginine deiminase (ADI) pathway genes that aid in the conversion of arginine to ATP. They sequenced the full ADI operon from the GL100 Mccp strain and the highest divergence was noted in a region coding for arcD. A typing method with improved resolution based on MLSA has been developed by Manso-Silván et al. [27] and the polymorphic region of the H2 locus was complemented with seven new loci from twenty-five strains showing polymorphisms (53 polymorphic positions) among the genome sequences (15 sequences). Whole genome sequencing of the M1601 Mccp strain has been performed by Chu et al. [21] on the Illumina GA platform using one flow cell lane with 36-cycle paired-end chemistry and gene prediction analyses performed by Glimmer3.0. They noted 961 open reading frames (ORFs) in the genome with an average gene length of 824 bp. Two rRNA operons, 30 tRNA genes, and one tmRNA gene were noted. A gene cluster involved in the synthesis of the capsule has been identified in the genome. This is comprised of genes encoding putative glycosyltransferases and UTP-glucose-1-phosphate uridylyltransferase. Both the *glpF*, *glpK*, and *glpD* gene cluster and the *gtsA*, *gtsB*, and *gtsC* gene cluster have roles in glycerol transport and the production of hydrogen peroxide. Genes encoding a lipoprotein precursor (*lppB*) and hemolysin A (*hlyA*), which have been responsible for pathogenicity in another *Mycoplasma,* have also been noted [21,81]. Additionally, eight genes encoding putative variable surface proteins have been identified and are responsible for antigenic variation in this *Mycoplasma* strain [21]. 

Dupuy and Thiacourt [79] studied the genome sequence of Mccp strain 9231-Abomsa and Falquet et al. [80] studied the virulent Mccp strains, F38 and ILRI181. A large-scale genomic approach based on NGS technologies has been applied by Dupuy et al. [68]. It overcame limitations of previous multilocus sequence typing that relied on house-keeping genes or polymorphic loci targeting by focusing on non-house-keeping genes and covering less than 1% of the genome [68]. Four genes did not exist in the Mccp genome, including *guaC*, *gntR*, *suk*, and *bgl*, while *dnaC* was duplicated. A subset of 57 genes, including 47 coding sequences and 10 pseudogenes, were evenly distributed along the chromosome of the 9231-Abomsa strain. Previous genome sequencing of different Mccp strains resulted in descriptions of various genes [21,79,80], but virulence factors of this important pathogen were not properly deciphered. While sequencing the genome of Mccp strain M1601, Chen et al. [20] identified 915 genes, occupying 90.27% of the genome, of which 713 were protein-coding genes (excluding 163 pseudogenes). They noted 26 genes (including ones for an adhesion protein, two capsule synthesis gene clusters, two lipoproteins, hemolysin A, *ClpB*, and proteins involved in pyruvate metabolism and cation transport) which were potential virulence factors and believed to be putative determinants associated with microorganism virulence. Two transporter systems, including the ATP-binding cassette (ABC) transporter and phosphotransferase, and two secretion systems, which are Sec and signal recognition particle (SRP) pathways, were also noted [20]. The Mccp genome contains a gene cluster (XDU01000075, XDU01000076, XDU01000814, and XDU01000816) which is involved in capsule synthesis. The genes comprising these clusters encode glycosyltransferase, UTP–glucose-1-phosphate uridylyltransferase, and diacylglyceryl transferase [20]. Clp genes noted in Mccp genome include ClpC, ClpB, and *clpB.* ClpC, an ATPase, plays an important role in cell adhesion and invasion. *ClpB* is concerned with stress responses, acting as a chaperone to avoid protein clumping and helping in the restructuring of denatured proteins. *ClpB* may be related to the virulence of Mccp. One ClpB gene (XDU01000405) was identified in the genome of Mccp and resembles the ClpB protein of *Listeria monocytogenes*. Thus, ClpB may be a virulence factor of Mccp [20]. Further, the Mccp genome contains genes for variable surface proteins, VmcC lipoprotein (XDU01000612), P60 surface lipoprotein (XDU01000037), and hemolysin A (XDU01000067), which have roles in antigenic variation, survival, and virulence, respectively [20]. Four PDH complex genes and two lipoate-protein ligase A (*lplA*) genes were identified and play pivotal roles in pyruvate metabolism. Thus, they may be essential for disease pathogenesis and hence act as virulence factors [20]. Two gene clusters *glpF–glpK–glpD* (XDU01000242, XDU01000243, and XDU01000244) and *gtsA–gtsB–gtsC* (XDU01000486, XDU01000487, and XDU01000488), which are important for glycerol metabolism, have been identified in the Mccp genome. They also act as virulence factors [20]. Three magnesium transporter genes (XDU01000099, XDU01000796, and XDU01000848), one potassium transporter TrkA (XDU01000743), and one sodium transporter (XDU01000742) were noted in the Mccp genome and are involved bacterial virulence [20]. Recently, genes encoding the complete glycerol uptake and hydrogen peroxide production metabolic pathways have been identified in Mccp [19]. Comparative genomic analysis of Mccp strain 87001 has revealed that its genome consists of a single circular chromosome having 1,017,333 bp in length and encoding for 898 open reading frames (ORFs) averaging 944 bp in length [82]. The study showed that there are 58 virulence genes in Mccp strain 87001 including variable surface lipoproteins, hemolysin A, and P60 surface lipoprotein. Of these, eight virulence genes and four extracellular genes that remained conserved over years are believed to be potential vaccine candidates. 

## 7. Other Possibilities

Since Mccp, like other mycoplasmas, have cytoplasmic and membranous structures, these structures can be explored for possible roles in virulence and their antigenic moieties can also be investigated. Cellular structural components, such as the capsule and proteins, or their individual components, such as polysaccharides and peptides, are under current investigation for their antigenic roles. Such exploration needs to be extended to include other components. In addition to the capsule, the plasma membrane, ribosomes, nuclear material, enzymes, and metabolites may have potential roles in virulence and antigenicity [20,42]. Utilization of hyperimmune serum against CCPP needs to be evaluated for prophylaxis as it can boost immunity and may be therapeutic during outbreaks, since it can provide an instant antibody that neutralizes pathogenic mechanisms [15,17,19,62,83]. However, there are limitations of this approach since CCPP usually occurs in outbreaks where numerous animals are affected at the same time. Raising such large quantities of hyperimmune serum may not be a feasible option, although there are some intensive farming prospects for this kind of intervention.

Mccp belongs to the *Mycoplasma mycoides* cluster, which has two groups, *M. mycoides* and *M. capricolum*. The mycoides group includes *Mycoplasma mycoides* subsp. *mycoides* small colony (SC) (MmmSC), *M. mycoides* subsp. *mycoides* large colony (LC) (MmmLC), *M. mycoides* subsp. *capri* (Mmc), and *Mycoplasma* species bovine group 7 (*Mycoplasma* sp. strain PG50). The capricolum group consists of *M. capricolum* subsp. *capricolum* (Mcc) (formerly *M. capricolum*) and *M. capricolum* subsp. *capripneumoniae* (Mccp) (formerly *Mycoplasma* sp. strain F38) [5,84,85]. However MmmLC and Mmc have been grouped in single subspecies. These six members of the mycoides cluster are antigenically related, as they share some common antigens [21,42,67,85,86,87]. Hence, cross-reactions are frequently noted in diagnostic tests [54,55]. In other words, antigens identified or evaluated in other members of the mycoides cluster can be explored in Mccp also. Some of the antigens recognized in mycoplasmas are pyruvate dehydrogenase [88], surface lipoproteins LppA (p72) [89], LppB, LppC, and LppQ, the surface protein Vmm [88,90,91], dihydrolipoamide acetyltransferase [88], P30, P40 [92,93], galactofuranose [94], dihydrolipoyl dehydrogenase, MbovP579 [95], phosphate acetyltransferase, substrate binding protein (OppA), permease (OppC), ATP-binding protein (OppF) [96], phosphopyruvate hydratase, variable surface membrane proteins family (Vpma) [97], adenine phopshoribosyltransferase, Pts-G (glucose phosphotransferase system permease) [98], transketolase, translation elongation factors G and Ts [88], recombinant E1 beta subunit of the pyruvate dehydrogenase complex [99], FMN-dependent NADH-azoreductase, peptide methionine sulfoxide reductase, inorganic diphosphatase, and trigger factor [88]. Further, Mccp has two distinct biochemical groups based on different metabolizing abilities for organic acid; and glucose and glycerol indicating virulence mechanism for pathogenesis [100].

In our previous review, we summarized possible future therapeutic targets for *Mycoplasma* infections [42]. These targets include structural units, proteins, enzymes, metabolites, or genes and other genetic material. The same can be explored for antigenic roles. Among the many antigens or virulence factors that are potential targets, enzymes, proteins, DNA or related structures, metabolites, and oxidative radicals should be considered. Enzymes along with their homology include DNA gyrase and/or DNA topoisomerase IV (96.1%), lysine-tRNA ligase (92.5%) [16,101]; glycolytic enzymes, e.g., pyruvate dehydrogenases A to C (PDHA-C) (99.1–99.7%), phosphoenolpyruvate-protein phosphotransferase (99%), glyceraldehyde-3-phosphate dehydrogenase (GapA) (96.5%), lactate dehydrogenase (Ldh) (98%), phosphoglycerate mutase (Pgm), pyruvate kinase (Pyk), transketolase (Tkt) (99.1%) [102]; nucleoside-catabolizing enzymes, e.g., nucleoside phosphorylase (NP)-II class PyNPs (pyrimidine NPs), NP-II class thymine phosphorylases, and NP-I class uridine phosphorylases [103]; pyrimidine nucleoside phosphorylase [104]; and metabolic enzymes, e.g., glycerophosphodiesterase [105], and glycerol-3-phosphate oxidase (93.8%) [106]. Proteins include surface-displayed proteins, e.g., PDHB, GapA, and Pyk (>99%) [102]; glycerol and phospholipid transporters, e.g., GlpU transport protein MPN421, proteins MPN076, and MPN077; glycerol facilitator GlpF [107], lipid-associated membrane proteins [108,109], lipoprotein MslA [110], triacylated lipoproteins N-ALP1 and N-ALP2 [111], and cytoplasmic and lipid-associated membrane proteins [112]. Some specific proteins with higher percentage of homology are cytosol aminopeptidase family, catalytic domain protein (98.2%), aldehyde dehydrogenase (NAD) family protein (98.3%), putative RNA polymerase sigma factor RpoD (94.5%), glutamyl-tRNA (Gln) amidotransferase subunit A (100%). DNA-related targets include mutant genes [113], targeted chromosomal knockouts [114], the QRDR region (DNA gyrase and topoisomerase IV) [101], 30S ribosomal subunit [101,115], 50S ribosomal subunit [101,115], domains II and V of the 23S rRNA gene, and the ribosomal protein L4 and L22 genes [112]. Metabolites that are possible virulence factors include hydrogen peroxide [106], glycerol [106,116], or oxidative radicals [42].

**Figure 1:**
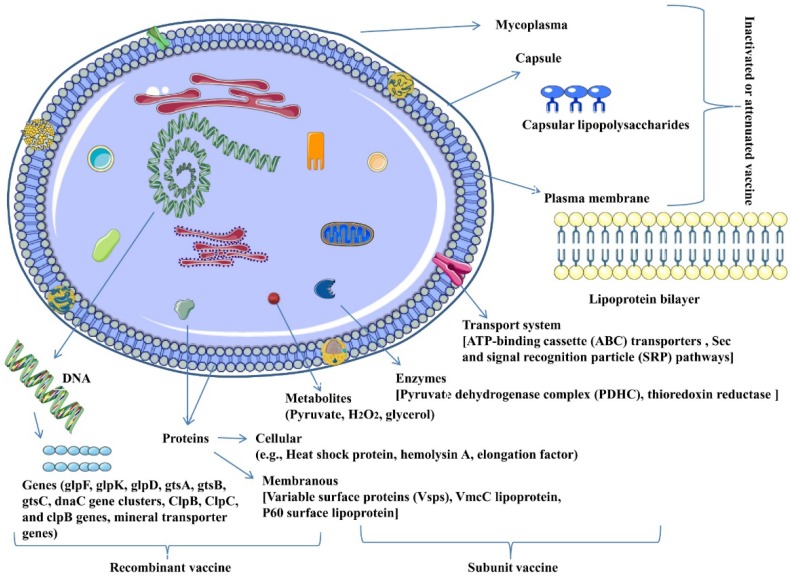
Vaccine candidates of *Mycoplasma capricolum* subspecies *capripneumonia.*

The various vaccine candidates for Mccp are shown in Figure 1.

## 8. Clues from Other Novel Vaccines

Numerous attempts have been made for vaccine development against *Mycoplasma* microorganisms, exploring DNA, proteins, or capsular polysaccharides for effective control. Novel approaches include recombinant protein or DNA vaccines [117,118], subunit vaccines [119,120], nanoparticle-based vaccines [121,122], plant-based or edible vaccines [123,124], and cytokine or TLR-based vaccines [125,126,127].

Protein-based vaccines are popular as proteins are convenient to isolate, evaluate, and usually provide an acceptable antigenic and immune response. As previously discussed, outer Mccp membrane proteins of the pyruvate complex PDHA, PDHB, and PDHC have been expressed in *E. coli* and explored as recombinant protein vaccine candidates. They have been expressed in vivo, show potential antigenicity, provide suitable reactogenicity, and elicit good humoral immune responses [17]. Hence, these entities which are involved in energy metabolism processes may find a role in vaccine development. Membrane protein P48 has been evaluated as a vaccine candidate in *Mycoplasma agalactiae* [128]. Lipoprotein LppA, which is a 62 kDa major surface protein found in a few members of the mycoides cluster, including *Mycoplasma mycoides* subsp*. capri* and *Mycoplasma mycoides* subsp*. mycoides,* has been explored as a vaccine candidate [117].

DNA vaccines are gaining importance in due to their ability to induce de novo production of antigens in the same manner as live vaccines. Both cellular and humoral immune responses are produced by DNA vaccines, which are stronger and last longer due to stimulation of immunological memory [117,129]. Chen et al. [130] designed DNA vaccines against *Mycoplasma hyopneumoniae* by constructing a pcDNA3/P42 plasmid. This carries the heat shock protein P42 gene and provides both cellular and humoral immune responses [130]. These purified recombinant antigens and two DNA vaccines, pcDNA3.1(+)/HSP70212-601 and pcDNA3.1(+)/MnuA182-378, of *Mycoplasma hyopneumoniae* have provided a satisfactory humoral immune response. Membrane lipoproteins have the potential to be targeted as DNA vaccine candidates against mycoplasmas [128]. Gene MCAP_0369/dnaK, which codes for heat shock protein 70 in *Mycoplasma capricolum* subsp. *capricolum* ATCC 27343, and gene MMS_A1021, which codes for DNA topoisomerase in *Mycoplasma mycoides* subsp. *mycoides* SC type, PG1 and Gladysdale strains, can be used as vaccine candidate. However, the membrane proteins have more promise than the cellular proteins [16]. Adhesion proteins in some mycoplasmas have been used as candidates for novel subunit vaccines. Cytoadhesion genes *mgc1* and *mgc2,* which code for adhesion proteins in *Mycoplasma gallisepticum*, have been cloned into plasmid vectors and transformed into *E. coli* [119]. Immune responses to P46, HSP70, and MnuA antigens (P46102-253, HSP70212-601, and MnuA182-378) were studied as recombinant subunits or DNA vaccines [120]. The recombinant E1 beta subunit of the PDHB complex in *Mycoplasma bovis* has been explored as an immunogenic protein and has shown excellent immune reactivity [98]. Hence, it can also be investigated as a vaccine candidate. Recombinant vaccine constructs using the TM-1 protein of *Mycoplasma gallisepticum* (pBH-S1-TM-1-EGFP) have been studied by Zhang et al. [118].

Since vaccine adjuvants like saponin or alum are having deleterious side effects, novel moieties are being investigated. Nanoparticles have shown a promising role in enhancing and modulating antigen immunogenicity, thereby inducing a strong, protective, and long-lasting immune response. Furthermore, adjuvants have shown characteristics related to promoting cell recruitment, antigen-presenting cell activation, as well as cellular (cytokine production) and humoral (antibody production) immune responses [121]. Recently, mesoporous silica nanoparticles have been effective as adjuvants by eliciting better cellular and humoral immune responses in recombinant *Mycoplasma hyopneumoniae* antigen vaccines [122]. Since there have always been concerns related to adverse side effects when using chemical or metal-based adjuvants, novel natural products have been investigated for their possible role as vectors in vaccines. Plant-based vector vaccines have potential uses in animal science [123,131]. Plants, both natural and transgenic, can produce and deliver antigens that have potential immunogenicity and hence, can serve as vaccines [124]. Recombinant proteins expressed in plants can be exploited as edible vaccines [123]. However, there have been adverse reactions with the use of these plant adjuvants too [132]. Therefore, biological adjuvants, such as cytokines or Toll-like receptors (TLRs) or their agonists, are being evaluated [125,126,133]. They are believed to be immune potentiators, as they activate immune cells directly [123,134]. TLR2 is known to sense lipopeptides in mycoplasmas, so it can act as a potent adjuvant and immunotherapeutic agent [127,135].

## 9. Conclusions and Future Prospects

CCPP vaccine development has evolved from the utilization of crude preparations, such as pleural fluid and lung extracts or homogenates, which resulted in effective protection but showed disease occurrence and lacked scientific standards. This was followed by the application of whole cultures, live attenuated, inactivated, and killed vaccines. Although live cultures of organisms were providing strong immunity, the risk of infection persisted. Attenuated, inactivated, and killed vaccines proved beneficial, provided immunity for more than a year, and were stable. Hence, they gained popularity and were commercialized. However, larger dose requirements, high production cost, adverse reactions to adjuvants, and problems associated with whole organisms raised concerns. Novel, safe, effective, and economical adjuvants are now under consideration. Subcellular antigenic components of Mccp have been recognized. Current research in CCPP vaccine development has unraveled immunogenic polysaccharides, proteins (both membranous and cellular), enzymes, genes, or metabolic pathways as better prophylactic possibilities using advanced technologies, including SDS-PAGE, immunoblotting, MALDI-TOF mass spectrometry, tandem mass spectroscopy, FPLC, and genomic approaches. MLSA, NGS, gene expression, and recombinant protein expression are all helpful genomic approaches to consider. These new strategies will not only identify antigenic proteins and virulent genes with vaccination potential, but will also help to overcome the limitations of past vaccines.

Despite the fact that various vaccine forms have been developed, applied, and some were proven effective, the disease has not been prevented or controlled, with endemic areas threatening disease-free areas. Many countries continue to have insufficient vaccination of their goat population, preventing the control of disease [31]. Hence, there is an opportunity for future development and application of CCPP vaccines. There is a need for proper analyses, identification, exploration, and evaluation of potent antigens, which are both safe and effective to use as vaccines. Novel proteomic, genomic, and metabolomic technologies can unravel Mccp cellular and molecular features, thus deciphering candidate antigens for vaccine development.

The countries where CCPP is endemic are usually developing countries, while the most vulnerable countries are disease-free and more likely to contract CCPP from neighboring affected countries. Therefore, it is clear that efforts to curb this disease need to be strengthened. Although a few vaccine development efforts have been started in developing countries, with international collaboration, and are being refined from time-to-time, the limited funding hampers development and renders researchers helpless. The financial funding from international sources, such as OIE, and the technical guidance from experts in *Mycoplasma* biology can boost such research for achieving better outcomes. 

## Figures and Tables

**Table 1:** Various vaccine candidates against *Mycoplasma* microorganisms.

*Mycoplasma*	Vaccine Type/Candidate	Remarks	Commercial Availability	Dose, Route	Reference
*Mycoplasma capricolum* subspecies *capripneumoniae* (Mccp)	Lung extract or pleural fluid from affected animals	Crude type of vaccination	Commercially not available	Subcutaneously	[46]
Mccp strain F38	Attenuated/passaged broth culture	Culture medium-based vaccination	Commercially not available	10 mL (10^9^ CFU), intratracheal route	[47]
Sonicated antigens of the Mccp strain F38	Inactivated or attenuated with incomplete Freund’s adjuvant (IFA), emulsified with aluminum hydroxide or phosphate buffered saline (PBS)	Antigen incorporated in IFA provided solid immunity to the challenge	Commercially not available	Subcutaneously	[10]
Lyophilized Mccp strain F38	Lyophilized vaccine	Challenged goats developed complete immunity to experimentally induced-CCPP	Commercially not available	Minimum dose 0.15 mg, subcutaneously	[7]
Strain F38 of *Mycoplasma* in saponin	Inactivated or attenuated Mccp vaccines	Provided immunity for more than 12 months, can be stored at 4 or 22 °C for 14 months	Commercially available. Cost about 100 USD	Dose of 0.15 mg optimum, subcutaneously	[11]
Sonicated antigens of the Mccp strain F38	Inactivated or attenuated with Freund’s incomplete adjuvant (IFA), saponin, aluminium hydroxide gel or PBS	Saponin and IFA were similar in their immune potentiating ability and were superior to aluminum hydroxide	Commercially not available	Subcutaneously	[63]
Mccp strain F38	Formalinized *Mycoplasma* culture	Optimum age for vaccination beyond 10 weeks of age	Commercially not available	1 mL per goat	[50]
Mccp strain F38	Inactivated Mccp strain F38-saponin vaccine	3 mg of saponin inactivates 1 mL of sonicated protein (2 mg/mL). 100% protection against natural CCPP	Commercially available. Cost about 100 USD	1 mL per goat, subcutaneously	[8]
Mccp strain F38	Polysaccharide vaccines	Humoral immune response	Vaccine candidates. Commercially not available	Vaccination potential not evaluated	[51]
Mccp strain F38	Lyophilized, saponin-killed vaccine, field vaccine	100% protection against mortality and 95% protection against clinical disease	Commercially available. Cost about 100 USD	Single dose of 0.15 mg, subcutaneously	[52]
Mccp strain F38	Epitope on a surface-exposed polysaccharide antigen	Detected by monoclonal antibody (mAb) WM-25	Vaccine candidate. Commercially not available	Antiserum raised against this epitope caused growth inhibition and agglutination. Developed immune response in goats	[64]
Mccp strain F38	Crude or membrane protein antigens	Identified by spotting on nitrocellulose using immunobinding	Vaccine candidate. Commercially not available	Standardized 0.5 mg protein/ml and finally 4 out of 60 specific antigens were identified by mAbs. Vaccination potential not evaluated	[53]
Mccp strains (G22, G94/83, G108/83, and G280/80	Specific integral membrane surface protein (p24)	Identified with the help of mAb E8-18	Vaccine candidate. Commercially not available	Vaccination potential not evaluated	[55]
Mccp strain 19/2	Subunit fractions of Mccp	Inhibitory effects as vaccines were noted	Vaccine candidate. Commercially not available	Vaccination potential not evaluated	[56]
Mccp strain 19/2	Capsular polysaccharide (CPS)	Prophylactic ability evaluated	Vaccine candidate. Commercially not available	Vaccination potential not evaluated	[15,56,57]
Mccp strain 19/2	Immunodominant core proteins (108, 70, 62 kDa)	Basis for recombinant protein for prophylactic vaccine	Vaccine candidate. Commercially not available	Vaccination potential not evaluated	[18]
Mccp Kenyan isolate (F38)	Saponin-adjuvated vaccines versus Montanide ISA 50-adjuvated vaccine	Montanide ISA 50-adjuvanted vaccines are safe, effective and show compatibility	Commercially available. Cost about 100 USD	1 mL/animal of saponin and Montanide ISA 50 adjuvated vaccines and 2 mL of MCCP and *Bacillus anthracis* adjuvanted with saponin. 0.2 mg of *Mycoplasma* protein per animal and 3 mg/dose of saponin. Subcutaneous injection into right thoracic wall	[12]
Mccp F38 strain	Inactivated F38 vaccine, aluminum hydroxide terpene vaccine	Booster 1 month, immunity short lived, kids vaccinated at above 12 months age	Commercially available. Cost about 100 USD	Recommended as vaccination	[58]
Kenyan isolate of Mccp	Live vaccines	Absence of any post-vaccination reaction, early appearance and longer persistence of antibodies. Chance of disease outbreaks	Commercially available. Cost about 100 USD	Dose of 10^5^ Mccp candidate live vaccine. Subcutaneously into right thoracic wall	[59]
F-38 Kenyan strain of Mccp	Inactivated whole culture vaccines	61.1% goats seroconverted, higher antibody titer in young and adult goats than older ones	Commercially available. Cost about 100 USD	1 mL/goat subcutaneously into neck region	[60]
Mccp Kenyan isolate	Inactivated whole culture vaccine	Equally safe and immunogenic as other vaccines but easy production, requires less time and is not capital investment intensive	Commercially available. Cost about 50–100 USD	2 mL subcutaneously	[61]
Mccp (local isolate)	Saponin-inactivated vaccine	14 month-shelf life and 12 month-immunity. Adverse reactions and incompatibility issues of saponin adjuvants, not recommended in pregnant animals	Commercially available. Cost about 100 USD	1 mL per goat (0.15 mg of freeze-dried-Mccp protein and 3 mg of saponin in a dose of 1 mL) subcutaneously	[2]
Mccp (strain M1601, ILRI181, 9231-Abomsa)	Proteins (both membranous and cellular)	Heat shock protein 70 (HSP70), elongation factor G, glutamyl-tRNA amidotransferase subunit A family catalytic domain protein, aldehyde dehydrogenase (NAD) family protein, thioredoxin reductase (NADPH), elongation factor Tu and peptidase M24 family	Vaccine candidate. Commercially not available	Vaccination potential not evaluated	[13,16,17]
Mccp (strain M1601, 9231-Abomsa)	Proteins, peptides	Variable surface proteins (Vsps), VmcC lipoprotein, P60 surface lipoprotein, hemolysin A, specific peptides	Vaccine candidate. Commercially not available	Vaccination potential not evaluated	[13,17,20,21]
Mccp (strain M1601, ILRI181, strain 9231-Abomsa)	Genes	16S rRNA genes, H2 locus and arginine deiminase (ADI) operon, glpF, glpK and glpD gene cluster, and gtsA, gtsB, and gtsC gene cluster, dnaC, adhesion protein gene, capsule synthesis gene clusters, lipoproteins, hemolysin A, ClpB, ClpC, and *clpB* genes, magnesium transporter genes (XDU01000099, XDU01000796, XDU01000848), potassium transporter TrkA (XDU01000743) and sodium transporter (XDU01000742) genes	Vaccine candidate. Commercially not available	Vaccination potential not evaluated	[20,21,65,66,67,68]
Mccp (strain M1601, ILRI181)	Enzymes	Pyruvate dehydrogenase complex (PDHC), transketolase, phosphoenolpyruvate protein phosphotransferase, L-lactate dehydrogenase, cytosol aminopeptidase, L-α-glycerophosphate oxidase (GlpO)	Vaccine candidate. Commercially not available	Vaccination potential not evaluated.	[16,19]
Mccp (strain M1601, ILRI181)	Metabolic pathways	Pyruvate metabolism, transporter systems [ATP-binding cassette (ABC) transporters and phosphotransferase], secretion systems [Sec and signal recognition particle (SRP) pathways], glycerol uptake, hydrogen peroxide production	Vaccine candidate. Commercially not available	Vaccination potential not evaluated.	[17,19,20]
